# Robust, practical and comprehensive analysis of soft compression image coding algorithms for big data

**DOI:** 10.1038/s41598-023-29068-z

**Published:** 2023-02-02

**Authors:** Gangtao Xin, Pingyi Fan

**Affiliations:** 1 The Department of Electronic Engineering, Tsinghua University, Beijing, 100084 China; 2 The Beijing National Research Center for Information Science and Technology, Tsinghua University, Beijing, 100084 China

**Keywords:** Applied mathematics, Information technology

## Abstract

With the advancement of intelligent vision algorithms and devices, image reprocessing and secondary propagation are becoming increasingly prevalent. A large number of similar images are being produced rapidly and widely, resulting in the homogeneity and similarity of images. Moreover, it brings new challenges to compression systems, which need to exploit the potential of deep features and side information of images. However, traditional methods are incompetent for this issue. Soft compression is a novel data-driven image coding algorithm with superior performance. Compared with existing paradigms, it has distinctive characteristics: from hard to soft, from pixels to shapes, and from fixed to random. Soft compression may hold promise for human-centric/data-centric intelligent systems, making them efficient and reliable and finding potential in the metaverse and digital twins, etc. In this paper, we present a comprehensive and practical analysis of soft compression, revealing the functional role of each component in the system.

## Introduction

Compression and communication are key issues in the information age, which have been widely concerned and explored by academia and industry. Shannon’s information theory^1^ answers two fundamental questions for both tasks^2^: What is the ultimate data compression (answer: the entropy *H*), and what is the ultimate transmission rate of communication (answer: the channel capacity *C*). The first answer theoretically gives a lower bound on data compression for independent and identically distributed (i.i.d.) random variables. This paper sets out to study compression, especially when the data is a general random process.

Compression is to reduce the cost of representing data as much as possible while ensuring the reconstruction quality. It can greatly lessen the user’s demand and dependence on communication and storage resources. In a nutshell, the goal of compression is to represent more with less. However, in the literature, theoretical and modeling works for compression are mainly concentrated on streaming data. For data with more complex structures such as images, it is still a challenging issue.

**Figure 1:**
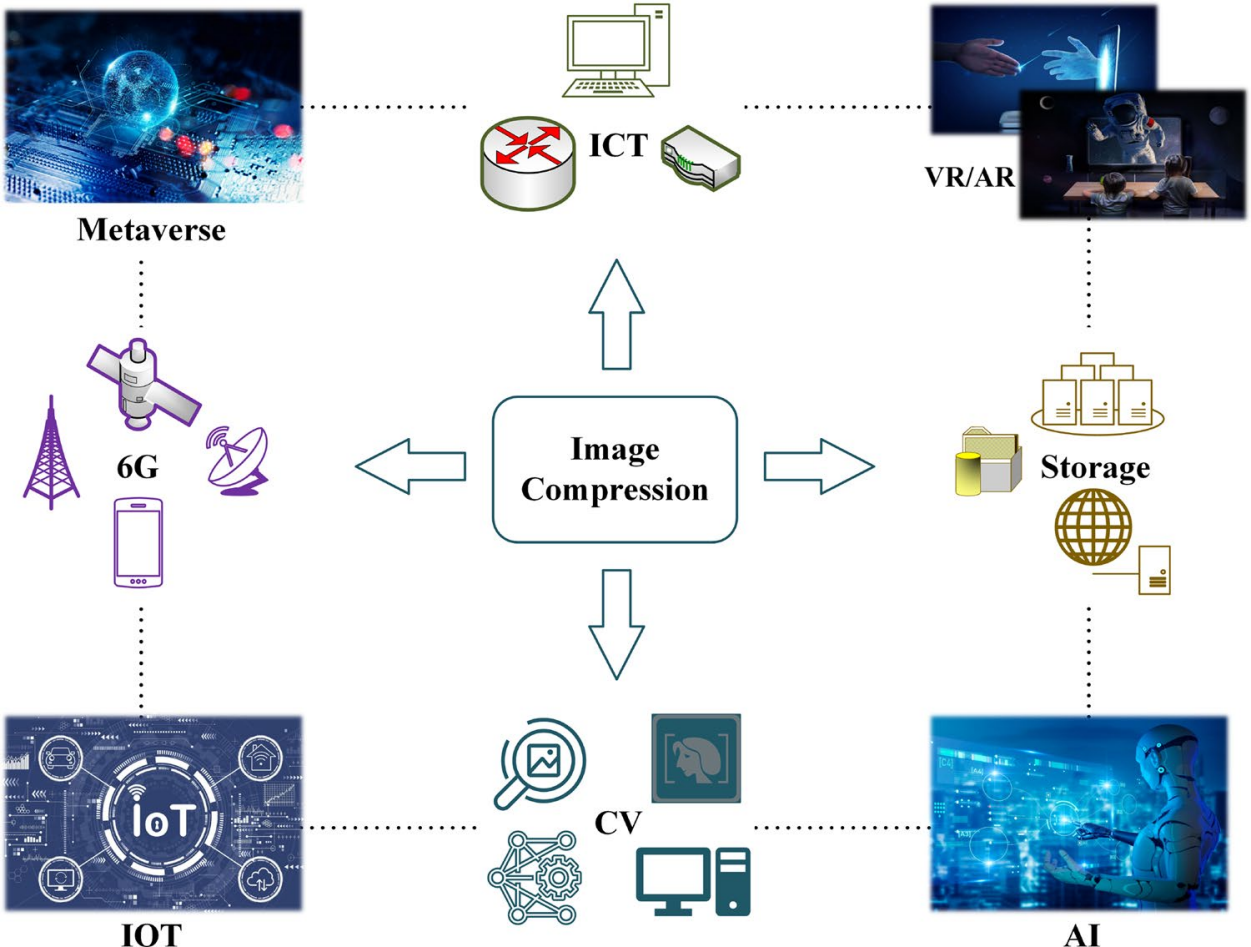
Image compression plays an important role in several scenarios, such as virtual reality (VR), augmented reality (AR), computer vision (CV), and the Internet of Things (IoT).

In general, data compression includes lossy and lossless paradigms. The former focuses on efficiency, while the latter is more concerned with quality. As an important meta-carrier, image compression directly affects the objective efficiency of the communication network and the subjective experience of clients. Image compression may play an important role in several fields, especially for metaverse and digital twins, such as virtual reality (VR), augmented reality (AR), computer vision (CV), and the Internet of Everything (IoE). Figure 1 shows the application of image compression in multiple scenarios. Moreover, data-driven image coding algorithms^3^ are expected to empower the Metaverse and 6G, becoming the backbone of next-generation intelligent technologies.

Recently, a novel data-driven and data-specific image lossless compression algorithm called *soft compression* was proposed in^4,5^. It has superior performance on the compression ratio, outperforming popular classical standards PNG and JPEG2000, as well as recent deep learning-based algorithms^6^. This paper focuses on a comprehensive analysis of the image coding algorithm with *soft compression*. We will provide a detailed description of the main properties of the image compression system in several scenarios.

### Preliminaries

In this paper, we mainly use several typical mutual information metrics and image quality assessment metrics to evaluate the characteristics of the soft compression algorithm. In this regard, we first give some definitions and explain some basic concepts which are used throughout the paper.

#### Mutual information

Mutual information is a measure of the amount of information that one random variable contains about the other^2^. In general, the smaller the mutual information, the weaker the correlation between two random variables from different event spaces. To date, mutual information has been effectively applied in many fields, such as telecommunications^7^ and machine learning^8,9^. In this study, we focus on three notable mutual information metrics, Shannon mutual information^1^, $\alpha$-mutual information^10^ and message importance measure loss^11^.

***(1) Shannon mutual information***

Shannon mutual information measures the reduction part in terms of the uncertainty for one random variable due to the knowledge of the other^2^. We use it to characterize the amount of information commonly shared between the two variables. The Shannon mutual information of two discrete random variables *X* and *Y* is defined by

1 $$\begin{aligned} I(X;Y) = \sum _{x \in \mathcal {X}} \sum _{y \in \mathcal {Y}} p(x,y)\log {p(x,y) \over {p(x)p(y)}}, \end{aligned}$$

where *p*(*x*, *y*) is the joint probability mass function of *X* and *Y*, and *p*(*x*) and *p*(*y*) are the marginal probability mass functions of *X* and *Y*, respectively.

***(2)*** $$\alpha$$ ***-Mutual information***

Rényi entropy and Rényi divergence^12^ evidence a long track record of usefulness in information theory and its applications^10^. In the literature, there are several possible ways to generalize Shannon mutual information in a similar way, notably by Suguru Arimoto^13^, Imre Csiszár^14^ and Robin Sibson^15^. We use Sibson’s definition for the measure. The Rényi mutual information of order $\alpha$, where $\alpha \ge 0$ and $\alpha \ne 1$, is defined as

2 $$\begin{aligned} I_{\alpha }(X;Y)= {\alpha \over {\alpha -1}} \log \sum _{y \in \mathcal {Y}} \bigg (\sum _{x \in \mathcal {X}} p(x) p^{\alpha }(y\vert x) \bigg )^{1 \over \alpha }. \end{aligned}$$

where $p(y \vert x)$ is the conditional probability of *Y* given *X*.

***(3) Message importance measure loss***

Message importance measure (MIM) was first proposed in 2016^16^. It is an important metric to describe the message in the scenario of big data. Similar to the Shannon entropy and Renyi entropy, MIM is required to reveal the uncertainty of a random process and some related statistical characteristics. The following definitions are intended to introduce the message importance measure theory briefly.

##### Definition 1

For a discrete random variable *X*, the exponential expression of message importance measure (MIM) is given by

3 $$\begin{aligned} L(\omega ,X)=\sum _{x \in \mathcal {X}} p(x) e^{\omega \{ 1-p(x)\}}, \end{aligned}$$

where the parameter $\omega$ is nonnegative and $p(x)e^{\{1-p(x)\}}$ is viewed as the self-scoring value of event $X=x$.

##### Definition 2

The conditional message importance measure (CMIM) is given by

4 $$\begin{aligned} L(\omega ,X \vert Y) = \sum _{y \in \mathcal {Y}}p(y) \sum _{x \in \mathcal {X}} p(x \vert y)e^{\omega \{1-p(x \vert y)\}} . \end{aligned}$$

##### Definition 3

The message importance loss based on MIM and CMIM is given by

5 $$\begin{aligned} \Phi _{\omega }(X \Vert Y) = L(\omega ,X)-L(\omega ,X \vert Y). \end{aligned}$$

MIM loss has been used in several fields such as information compression, distribution estimation, anomaly detection, recommendation, and IoT^11,17^. The parameter in MIM can be selected to amplify the weights of certain elements according to the user’s preference^18^. In Sect. “Mutual information measure analysis”, we will use Shannon mutual information, $\alpha$-mutual information, and MIM loss to measure the mutual information between different components of an image.

#### Image quality assessment

The image quality assessment metric reveals the similarity between the distorted image and the original image. It measures the quality of an image from an observational and computational point of view. The similarity of two images, *P* and *O*, is measured as

6 $$\begin{aligned} n(f(P),f(O)) = \Vert f(P)-f(O) \Vert _2^2, \end{aligned}$$

where $f(\cdot )$ is the image embedding function mapping an image to a point in Euclidean space. The image embedding function $f(\cdot )$ is the essential part for finding the similarity between two images. Common image quality assessment metrics include peak signal-to-noise ratio (PSNR), structural similarity (SSIM), and image semantic similarity. They are usually computed over all pixels in both images. We use them to measure the reconstruction ability of the Decoder in the compression algorithm.

***(1) Peak signal-to-noise ratio (PSNR)***

The simplest and most widely used image quality assessment metric is the mean squared error (MSE). It is computed by averaging the squared intensity differences of the distorted and reference image. Let *P* and *O* denote a digital image with intensity levels in the range $[0, D-1]$, where *D* is a positive integer number that represents the pixel intensity level of images. The row and column dimensions are *M* and *N*. Let *P*(*i*, *j*) and *O*(*i*, *j*) be the intensity value on (*i*, *j*) in *P* and *O*, respectively. MSE is defined as

7 $$\begin{aligned} MSE = {1 \over MN} \sum _{i=0}^{M-1} \sum _{j=0}^{N-1} [P(i,j)-O(i,j)]^2. \end{aligned}$$

Peak signal-to-noise ratio (PSNR) is an assessment metric for the ratio between the maximum possible power of a signal and the power of corrupting noise that affects the fidelity of its representation. It is defined as

8 $$\begin{aligned} PSNR = 20 \cdot \log _{10} \left( {D-1 \over {\sqrt{MSE}}}\right) . \end{aligned}$$

In general, the larger the PSNR, the better the quality of the reconstructed image.

***(2) Structural similarity (SSIM)***

Objective methods for assessing perceptual image quality traditionally attempted to quantify the visibility of errors between a distorted image and a reference image using a variety of known properties of the human visual system^19^. The structural similarity index is a framework for quality assessment under the assumption that human visual perception is highly adapted for extracting structural information from a scene. It reveals the structural similarity between the reconstructed image and the original image. For images *P* and *O* one can obtain

9 $$\begin{aligned} l(P,O)&= { {2\mu _P \mu _O +c_1} \over {\mu _P^2 + \mu _O^2 + c_1}} \end{aligned}$$

10 $$\begin{aligned} c(P,O)&= { {2\sigma _{PO} +c_2} \over {\sigma _P^2 + \sigma _O^2 + c_2}} \end{aligned}$$

11 $$\begin{aligned} s(P,O)&= { {\sigma _{PO}+c_3} \over {\sigma _P \sigma _O + c_3}}, \end{aligned}$$

where $\mu$, and $\sigma ^2$ represent the mean intensity and variance of an image, respectively. $c_1$, $c_2$ and $c_3$ are some positive constants.

Finally, the three parts are combined to yield an overall similarity measure.

12 $$\begin{aligned} S(P,O)=[l(P,O)]^{\alpha } [c(P,O)]^{\beta } [s(P,O)]^{\gamma }, \end{aligned}$$

where $\alpha$, $\beta$ and $\gamma$ are positive constants. SSIM has three main characteristics:

- Symmetry: $$S(P,O)=S(O,P)$$
- Boundedness: $$S(P,O)\le 1$$
- Unique maximum: $S(P,O)=1$ if and only if $P=O$.

SSIM is between 0 and 1. The larger the SSIM, the smaller the difference between the distorted image and the original image, that is, the better the image quality. SSIM equals 1 when two images are exactly the same.

***(3) Image semantic similarity***

Image semantic similarity metric depends on the high-order image structure, which is usually context-dependent^20–22^. DL-based image similarity metrics encode high invariance and capture image semantics. For example, one can measure the distance of two images in VGG feature space as the perceptual loss^23^. Richard *et al.*^24^ introduced a perceptual similarity dataset and evaluated deep features across different architectures and tasks. The visual translation embedding (VTransE) network^25^ is an end-to-end and fully-convolutional architecture that consists of an object detection module, a differential feature extraction layer, and a novel visual translation embedding layer for classification. As a result, the model can place objects in a low-dimensional relation space and measure semantic similarity. The relationship detection model in^26^ learns a visual and a semantic module that maps features from the two modalities into a shared space, where matched pairs of features have to discriminate against those unmatched, but also maintain close distances to semantically similar ones.

### Relations to big data

Big data are becoming related to almost all aspects of human activity from just recording events to research, design, production, and digital services or product delivery, to the final consumer^27^. In a number of discussions, blog posts, and articles, big data are attributed to have such characteristics as Volume, Variety, Velocity, Value, and Veracity^28^. Volume refers to larger amounts of data being generated from a range of sources. Variety refers to using multiple kinds of data to analyze a situation or event. The velocity of data is increasing rapidly over time for both structured and unstructured data, and there’s a need for more frequent decision-making about that data. Value refers to the data of low-value density. Users are required to address the need to enrich raw and unprocessed data by extracting higher-level knowledge for exploitation across different scenarios. The Veracity dimension deals with data uncertainty due to various factors such as data inconsistencies, incompleteness, and deliberate deception^29^.

With the advancement of intelligent vision algorithms and devices, image reprocessing and secondary propagation are becoming increasingly prevalent. A large number of similar images are being produced rapidly and widely, resulting in the homogeneity and similarity of images. In other words, as a data carrier, images also have the characteristics of big data. This brings new challenges to image compression systems, which need to exploit the potential of deep features and side information of images.

Soft compression is a novel data-driven image coding algorithm with superior performance, exploiting several characteristics of big data. Firstly, its codebook is not artificially specified but generated by training on the image database, which reflects the ’Volume’ of big data. Secondly, soft compression can be adaptively updated as the data changes, so it is suitable for different situations and keeps up with the ’Velocity’ of big data. Finally, the most important point is that soft compression algorithms can extract higher-level knowledge and features to form a codebook, which takes advantage of big data’s ’Value’ characteristic.

### Paper outline

The main contributions of this work can be summarized as follows:

- We point out the relationship between soft compression algorithms and big data. Moreover, we discuss how it exploits characteristics of Volume, Velocity, and Value of big data for image coding.
- We do a comprehensive and practical analysis of image coding algorithms with soft compression. In addition, the system was deployed in several scenarios, consisting of mutual information measure analysis, function evaluation of each component, noise robustness, and data transmission.
- It lays the foundation and provides information for the deployment of the soft compression algorithm in real scenarios. All these observations may have a bearing on general image coding methods and will be expected to provide more insights into the future multi-media semantic communications and find promising applications in the metaverse and digital twins, etc.

We introduces the soft compression strategy in Sect. “Methods”. The remaining part has been divided into four themed chapters, discussing the main properties of the soft compression algorithm in several scenarios. These analyses are more close to the ground truth since they are based on real datasets and experimental results. Section “Mutual information measure analysis” analyzes the results of mutual information measure between two components of images. In Sect. “Function evaluation of each component”, we lay out the experimental results of reconstructed images when some information is lost, and reveal the robustness of the image compression algorithm. It summarizes the key characteristics of these components, reflecting the functional effect of each component for a complete image. Section “Robustness to noise” presents the findings of noise robustness, focusing on the impact of different components on the reconstructed image in the presence of noise interference. Section “Data transmission” is concerned with the actual data transmission, combing the source and channel to analyze the entire communication process of an image. Finally, we conclude the paper in Sect. “Conclusion”.

## Methods

Most studies in the field of image compression have only focused on one of theory or engineering. Soft compression closely unified theory and algorithm through shapes, which brought advantages for guiding image processing and compression. The term *soft compression* will be used to describe a novel image encoding and decoding algorithm based on data distribution.

For a digital image *P*, we use *M* and *N* to represent the length and width, respectively. Suppose *P* is divided into *c*(*t*) shapes $\{s_1,s_2,...,s_{c(t)}\}$, where $t=M \times N$ and $s_i$ is the *i*-th shape. Let $\mathcal {D}$ denote the shape database, then the mathematical representation of image *P* with soft compression is described as^4,30^

13 $$\begin{aligned} \min&~~\sum _{i=1}^{c(t)} [l(s_i)+l_p(x_i,y_i)] \end{aligned}$$

14 $$\begin{aligned}&\quad \text {s.t.}~~P=\sum _{i=1}^{c(t)}F_i(s_i) \end{aligned}$$

where $l(s_i)$ and $l_p(x_i,y_i)$ represent the length of the shape $s_i$ and corresponding location at $(x_i,y_i)$, respectively.

In soft compression, we use the codebook to map shapes to bits. It is a bijection that provides the encoder with the basis for converting an image into binary characters. Furthermore, the codebook is obtained in a data-driven manner, reflecting the distribution characteristics of image datasets. The generation of codebooks consists of two parts, image processing, and shape updating. The details are given as follows.

**Figure 2:**
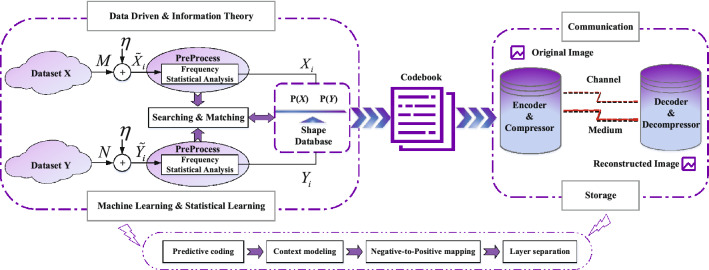
The structure of soft compression algorithm. It consists of the generation and use of the codebook. The former takes advantage of the characteristics of the data source, while the latter improves the compression performance.

1. Image processing. For a multi-component image, the first step is to obtain multiple single-component images through reversible component transformation, that is, color decorrelation for efficient image compression. For example, we convert the color space of an RGB image to YUV, which is given by
  15 $$\begin{aligned} \begin{aligned} \begin{pmatrix} Y \\ V \\ U \end{pmatrix} = \begin{pmatrix} \left\lfloor \displaystyle {\frac{R+2G+B}{4}}\right\rfloor \\ R-G \\ B-G \end{pmatrix} \qquad \begin{pmatrix} G \\ R \\ B \end{pmatrix} = \begin{pmatrix} Y-\left\lfloor \displaystyle {\frac{U+V}{4}}\right\rfloor \\ V+G \\ U+G \end{pmatrix} \end{aligned}. \end{aligned}$$
  Soft compression applies the same processing to each single-component image, the difference is only in the parameters of the algorithm. Let *I*(*x*, *y*) represent one of the single-component images. We use the general predictive coding and context modeling method such as^31^ and^32^ to get the predictive image $I_F(x,y)$. Then, the predictive difference image can be obtained by
  16 $$\begin{aligned} I_E(x,y) = I(x,y) - I_F(x,y). \end{aligned}$$
  To make the intensity value of $I_E(x,y)$ in the non-negative range, a mapping needs to be done. We have
  17 $$\begin{aligned} I'(x,y)=\left\{ \begin{aligned}&2I_E(x,y){} & {} I_E(x,y) \ge 0 \\&-2I_E(x,y)-1{} & {} I_E(x,y) < 0 . \\ \end{aligned} \right. \end{aligned}$$
  In addition, the last step is layer separation.
  18 $$\begin{aligned} I'_S(x,y)&= I'(x,y)~//~2^l \end{aligned}$$
  19 $$\begin{aligned} I'_D(x,y)&= I'(x,y)~\%~2^l , \end{aligned}$$
  where *l* is a constant that can be given empirically or experimentally. It divides $I'(x,y)$ into two parts, the shape layer $I'_S(x,y)$ and the detail layer $I'_D (x,y)$. The shape layer is regular, which reflects the coarse information of an image. On the other hand, the detail layer is chaotic, containing a lot of detailed information about an image, and seems to be random but has its nature related to the images.
2. Shape updating. The set of shapes with soft compression algorithm directly determines the performance and efficiency of encoding. Its acquisition is based on pre-definition, importance matching, and dynamic updating. Suppose *A* is an $m \times n$ matrix. $\varvec{u_i}$ and $\varvec{v_j}$ are vectors, representing the *i* row and *j* column of *A*, respectively. The matrix that can be used to design shapes satisfying the following constraints for compression
  20 $$\begin{aligned}&\Vert \varvec{u_i}\Vert _0 \ge {n \over 2}~~~~\forall ~1 \le i \le m \end{aligned}$$
  21 $$\begin{aligned}&\quad \Vert \varvec{v_j}\Vert _0 \ge {m \over 2}~~~~\forall ~1 \le j \le n . \end{aligned}$$
  We remove zeros from the matrix *A*, which is the initial candidate shape. They form a library of predefined shapes. Then, we sequentially match the shape library to images from multiple dimensions. The frequency of each shape is counted, and uncommon shapes are eliminated from the shape library. Moreover, shapes are given different importance depending on their frequency and size. Through iteration and dynamic updating, we can get the final shape database. Figure 2 illustrates the overall structure. Furthermore, we assign each shape a codeword based on its importance. They form a codebook that enables the transformation from two-dimensional pixels to one-dimensional bits.

The codebook is not only the basis of the coding but also builds a bridge between existing data and unknown data, acting as side information^33^. Encoders and Decoders also use codebooks to perform forward and reverse conversions from images to binary characters. When encoding, we first perform the same image processing operations on the image as in the codebook acquisition stage. For the shape layer, the shape database is used for matching, to represent an image as a combination of shapes. Then, we use the codebook to find the codeword corresponding to each shape to implement the mapping. On the other hand, we adopt the corresponding codebook to encode the detail layer. Compressed data is generated by concatenating the encoded results of multiple components. In this way, the entire encoding of an image is finished. Decoding is the reverse process, remapping the bits back to an image.

**Table 1:** Some statistics about compression ratio of image datasets with different compression methods (all methods are lossless compression).

Dataset	Statistic	Method			
		Soft compression	JPEG	PNG	JPEG2000
Malaria	Mean	**3.80**	2.53 *+50%*	2.83 *+34%*	3.56 *+6.7%*
	Minimum	2.35	1.77	1.87	2.50
	Maximum	6.88	4.41	7.46	8.92
	Variance	0.8058	0.3401	1.0340	1.2497
BCCD	Mean	**3.83**	2.24 *+71%*	2.49 *+54%*	3.56 *+7.6%*
	Minimum	3.34	2.06	2.18	3.11
	Maximum	4.57	2.54	2.87	4.20
	Variance	0.0535	0.0089	0.0166	0.0369
Melanoma	Mean	**3.44**	1.84 *+87%*	1.93 *+78%*	3.15 *+9.2%*
	Minimum	1.81	1.29	1.31	2.02
	Maximum	5.17	2.81	3.11	4.70
	Variance	0.4758	0.0596	0.0867	0.2365
FIRE	Mean	**4.71**	2.69 *+75%*	3.52 *+34%*	4.66 *+1.1%*
	Minimum	4.23	2.54	3.32	4.36
	Maximum	5.00	3.20	4.04	4.88
	Variance	0.0486	0.0180	0.0270	0.0250

Significant values are in bold and italics.

Table 1 illustrates the experimental results of soft compression algorithm for multi-component images and other classical systems on Malaria, BCCD, Melanoma, and FIRE datasets^34^. The statistics include mean, minimum, maximum, and variance of compression ratio. The results of Table 1 indicates that the average compression ratio with soft compression is higher than other image lossless compression methods. Through comparison, we can conclude that soft compression algorithm outperforms the popular classical benchmarks JPEG, PNG, and JPEG2000. There are several reasons why soft compression has become so dominant compared to other algorithms.

- **From hard to soft**. The codebooks are no longer artificially designed, but generated from data. Data-driven and data-specific make the codebook adaptive to the probability distribution of images. This is what *soft* means.
- **From pixels to shapes.** The basic unit of an image is the shape, rather than the pixel. An image is composed of combinations of different shapes. The transition from 1D to 2D brings the ability to compress images from multiple perspectives. As a result, the algorithm can simultaneously eliminate the coding and spatial redundancy, improving the compression performance of the encoder.
- **From fixed to random.** The arrangement of the basic units in an image is no longer a fixed order, but random. This greatly increases the flexibility of coding. The system can start and end encoding at any position in an image, which is no longer limited to a fixed paradigm. Moreover, it may serve higher-order images processing tasks such as object detection and semantic segmentation.
- **From coarse to fine**. During the execution of the algorithm, an image is divided into two layers: the shape layer and the detail layer. The shape layer outlines the main part of an image, and the detail layer contains a lot of detailed information. An implication of this is the possibility that the user’s perception of an image can be progressive. Consequently, the sender first transmits the shape layer when communicating, so that the receiver can have an overall understanding of the image. The detail layer is then transmitted to reconstruct a complete image. This allows the receiver to have a coarse-to-fine perception of the image, reducing waiting time and improving the user’s experience.

## Mutual information measure analysis

Soft compression algorithm decomposes an image into six different components: $Y_S$, $Y_D$, $U_S$, $U_D$, $V_S$, and $V_D$. These new components and the original image satisfy the bijective relationship. In this section, we investigate the mutual information between two components, especially the correlation before and after decomposition. The following ten tables are based on the DRIVE dataset^35^. The DRIVE dataset is obtained from a diabetic retinopathy screening program to study skin lesions. Table 2 illustrates the Shannon mutual information between two components. It shows the results of $I(Y;Y_S)$, $I(Y;Y_D)$, $I(U;U_S)$, $I(U;U_D)$, $I(V;V_S)$ and $I(V;V_D)$. We can observe that $I(Y;Y_D)$ is about an order of magnitude higher than $I(Y;Y_S)$. The same is true of $I(U;U_D)$ and $I(U;U_S)$, $I(V;V_D)$ and $I(V;V_S)$. It reveals that the detail layer has more information about the original image than the shape layer, that is, it needs to be described with more bits.

**Table 2:** Shannon mutual information measure.

	Y component		U component		V component	
	$I(Y;Y_S)$	$I(Y;Y_D)$	$I(U;U_S)$	$I(U;U_D)$	$I(V;V_S)$	$I(V;V_D)$
Value	0.02	0.21	0.01	0.15	0.01	0.13

The experimental results are obtained on the DRIVE dataset^35^.

The result in Tables 3 and 4 are similar to those reported in Table 5. It shows that under different mutual information metrics, the mutual information value of the shape layer and the original component is lower than that of the detail layer and the original component. The experimental results reveal the intrinsic characteristics of the shape layer and the detail layer. The shape layer is regular and reflects the main structured feature of an image. It can be completely represented with fewer bits. The detail layer is irregular and seems to be random, so it contains many details of an image. Intuitively, it needs to be represented by more bits.

**Table 3:** $\alpha$-Mutual information measure.

$\alpha$	Y component		U component		V component	
	$I_{\alpha }(Y;Y_S)$	$I_{\alpha }(Y;Y_D)$	$I_{\alpha }(U;U_S)$	$I_{\alpha }(U;U_D)$	$I_{\alpha }(V;V_S)$	$I_{\alpha }(V;V_D)$
0.1	0.0014	0.0355	0.0010	0.0219	0.0006	0.0167
0.9	0.0167	0.1936	0.0105	0.1427	0.0082	0.1218
2	0.0753	0.3378	0.0460	0.2470	0.0737	0.2436
10	0.4599	1.0528	1.7262	1.7438	1.4339	1.6494

The experimental results are obtained on the DRIVE dataset.

**Table 4:** Message importance measure loss.

$\omega$	Y component		U component		V component	
	$\Phi _{\omega }(Y \Vert Y_S)$	$\Phi _{\omega }(Y \Vert Y_D)$	$\Phi _{\omega }(U \Vert U_S)$	$\Phi _{\omega }(U \Vert U_D)$	$\Phi _{\omega }(V \Vert V_S)$	$\Phi _{\omega }(V \Vert V_D)$
0.1	3.8 $\times 10^{-5}$	1.4 $\times 10^{-3}$	4.1 $\times 10^{-5}$	1.4 $\times 10^{-3}$	1.6 $\times 10^{-5}$	1.1 $\times 10^{-3}$
1	8.6 $\times 10^{-4}$	3.2 $\times 10^{-2}$	9.0 $\times 10^{-4}$	3.0 $\times 10^{-2}$	3.7 $\times 10^{-4}$	2.4 $\times 10^{-2}$
2	4.3 $\times 10^{-3}$	1.5 $\times 10^{-2}$	4.4 $\times 10^{-3}$	1.4 $\times 10^{-2}$	1.9 $\times 10^{-3}$	1.1 $\times 10^{-2}$

The experimental results are obtained on the DRIVE dataset.

On the other hand, despite these recent findings about the role of the shape layer and the detail layer, the section that follows moves on to consider the function effect of each component for a complete image.

## Function evaluation of each component

The implementation of *communication* and *storage* is not always perfect. Images may lose some data in these processes, resulting in the decline of the visual effect. It is of scientific value to study the quality of reconstructed images when some information is lost. Moreover, it reveals the robustness of the image compression algorithm and may play a significant role in the abominable communication environment. On the other hand, we are concerned about the difference between the reconstructed image and the original image when one or more components are lost. The experimental results reflect the functional effect of each component for a complete image.

Table 5 illustrates the experimental results when one component is lost. We use PSNR and SSIM as assessment metrics to measure the difference between the reconstructed image and the original image. The order of PSNR value from low to high corresponds to the loss of $Y_D$, $V_D$, $U_D$, $Y_S$, $U_S$, and $V_S$. Similarly, the order of SSIM value from low to high corresponds to the loss of $Y_S$, $U_D$, $Y_D$, $V_D$, $U_S$, and $V_S$. The results reflect that $Y_D$ and $Y_S$ are the two main factors affecting the image assessment metric. *Y* contributes more to the whole picture than *U* and *V*. Moreover, the SSIM will be the worst if $Y_S$ is lost. It reveals the prominent role of $Y_S$ among six components.

**Table 5:** The quality of the reconstructed image when one component is lost.

Image quality assessment metric $^1$	Lost components						
	$\varnothing$ $^2$	$Y_S$	$Y_D$	$U_S$	$U_D$	$V_S$	$V_D$
PSNR	$\infty$	11.02	4.34	16.25	9.96	22.94	6.49
SSIM	1	0.31	0.45	0.81	0.44	0.88	0.45

The experimental results are obtained on the DRIVE dataset.

$^1$The difference evaluation between the reconstructed image and the original image.

$^2$It means no loss of components.

**Figure 3:**
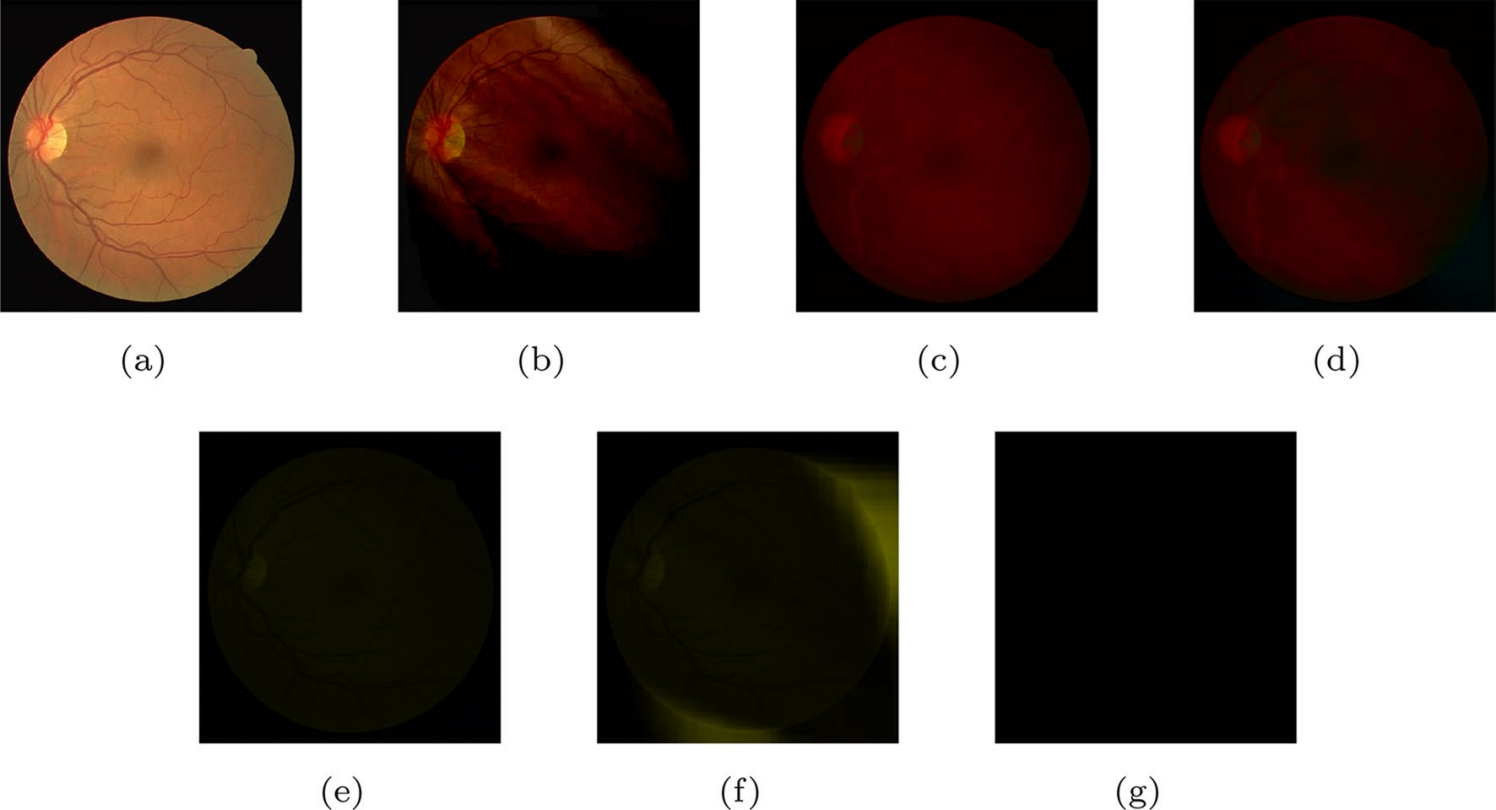
(**a**) The original image. (**b**) The reconstructed image without component $Y_S$. (**c**) The reconstructed image without components $Y_S$ and $Y_D$. (**d**) The reconstructed image without components $Y_S$, $Y_D$ and $U_S$. (**e**) The reconstructed image without components $Y_S$, $Y_D$, $U_S$ and $U_D$. (**f**) The reconstructed image without components $Y_S$, $Y_D$, $U_S$ and $U_D$. (**g**) The reconstructed image without components $Y_S$, $Y_D$, $U_S$, $U_D$, $V_S$ and $V_D$. See the text for more explanations.

Visual quality directly affects the subjective experiences of clients. Figure 3 illustrates that there is a gradual decline in visual quality as more components are lost. Figure 3a is an original image drawn from the DRIVE dataset. Figure 3b–f represent the reconstructed image with losing components $Y_S$, $Y_S Y_D$, $Y_S Y_D U_S$, $Y_S Y_D U_S U_D$ and $Y_S Y_D U_S U_D V_S$, respectively. Figure 3g shows the image without any components, that is, a blank image. During the transition from Fig. 3a–b, the image loses the skeleton information and becomes blurred. Moreover, the loss of $Y_D$ causes the image to lack the main structural feature, as shown in Fig. 3c. After losing $U_S$ and $U_D$, Fig. 3d and e become blurry further. Specifically, Fig. 3f retains some details about the color difference. Initial observations suggest that different components contain different information and make unique contributions to the whole image.

What can be clearly seen in Tables 5 and 6 is the dominance of the $Y_S$ component. The SSIM value of $Y_S$ is the largest when the image can only reserve one component. $Y_S$ represents the skeleton feature of an image. One possible implication is that the algorithm can be applied in an abominable communication environment. The transmitter can only send the component $Y_S$ to make the receiver have an overall understanding of the image. Then, the transmitter continues to send other components such as $Y_D$ and $U_S$. The receiver can fill these components into a complete image in turn. It can improve the objective quality of the communication network and the subjective experiences of users. The key features of these components can be summarized as follows:

1. *Y* contributes more to visual perception than *U* and *V*. This is consistent with the conclusions in engineering applications of image and video compression.
2. The shape layer component ($Y_S$, $U_S$ and $V_S$) and detail layer ($Y_D$, $U_D$ and $V_D$) represent coarse and fine information, respectively.
3. $Y_S$ represents the skeleton information of an image.
4. $Y_D$ represents the structural information.
5. $U_S$ and $V_S$ reflect the spatial hierarchy of an image.
6. $U_D$ and $V_D$ reveal the chroma of an image.

It is expected that such observed results can be used in future semantic communications.

**Table 6:** The quality of the reconstructed image when some components are lost.

Lost components	Image quality assessment metric $^1$	
	PSNR	SSIM
$Y_S$ $Y_D$	10.67	0.22
$U_S$ $U_D$	16.66	0.93
$V_S$ $V_D$	24.27	0.96
$Y_S$ $U_S$	9.77	0.31
$Y_S$ $V_S$	10.91	0.31
$U_S$ $V_S$	15.09	0.77
$Y_S$ $U_S$ $V_S$	9.63	0.31
$Y_D$ $U_D$ $V_D$	4.31	0.42
$Y_S$ $Y_D$ $U_S$ $U_D$	8.18	0.12
$Y_S$ $Y_D$ $V_S$ $V_D$	10.27	0.18
$U_S$ $U_D$ $V_S$ $V_D$	14.70	0.88
$Y_D$ $U_S$ $U_D$ $V_S$ $V_D$	4.19	0.42
$Y_S$ $U_S$ $U_D$ $V_S$ $V_D$	8.56	0.30
$Y_S$ $Y_D$ $U_D$ $V_S$ $V_D$	9.36	0.20
$Y_S$ $Y_D$ $U_S$ $V_S$ $V_D$	8.74	0.17
$Y_S$ $Y_D$ $U_S$ $U_D$ $V_D$	5.40	0.15
$Y_S$ $Y_D$ $U_S$ $U_D$ $V_S$	8.03	0.15

The experimental results are obtained on the DRIVE dataset.

$^1$The difference evaluation between the reconstructed image and the original image.

## Robustness to noise

The noise interference is unavoidably generated during storage and transmission. In this section, we explore the impact of different components on the reconstructed image under noise interference. For simplicity, we consider three popular noise interference models for images, i.e., salt and pepper noise, Gaussian noise, and exponential noise.

### Salt and pepper noise

The probability density function of salt and pepper noise is given by

22 $$\begin{aligned} p(z)=\left\{ \begin{aligned}&P_a,~~{} & {} z=a \\&P_b,~~{} & {} z=b \\&1-P_a-P_b,~~{} & {} \text {otherwise} \end{aligned} \right. \end{aligned}$$

where *z* represents the pixel intensity. $P_a$ and $P_b$ are probabilities. *a* and *b* are two constants. The intensity *b* will appear as a light dot in the image. Conversely, level *a* will appear like a dark dot^36^. What is striking in Table 7 is the dramatic difference between the two noise modes. The reconstructed image quality of mode I is significantly higher than that of mode II. It shows that there has been a sharp decline in the image quality when the noise changes from dark dots to light dots. The results reveal that all components are sensitive to light dots and more robust to dark dots.

**Figure 4:**
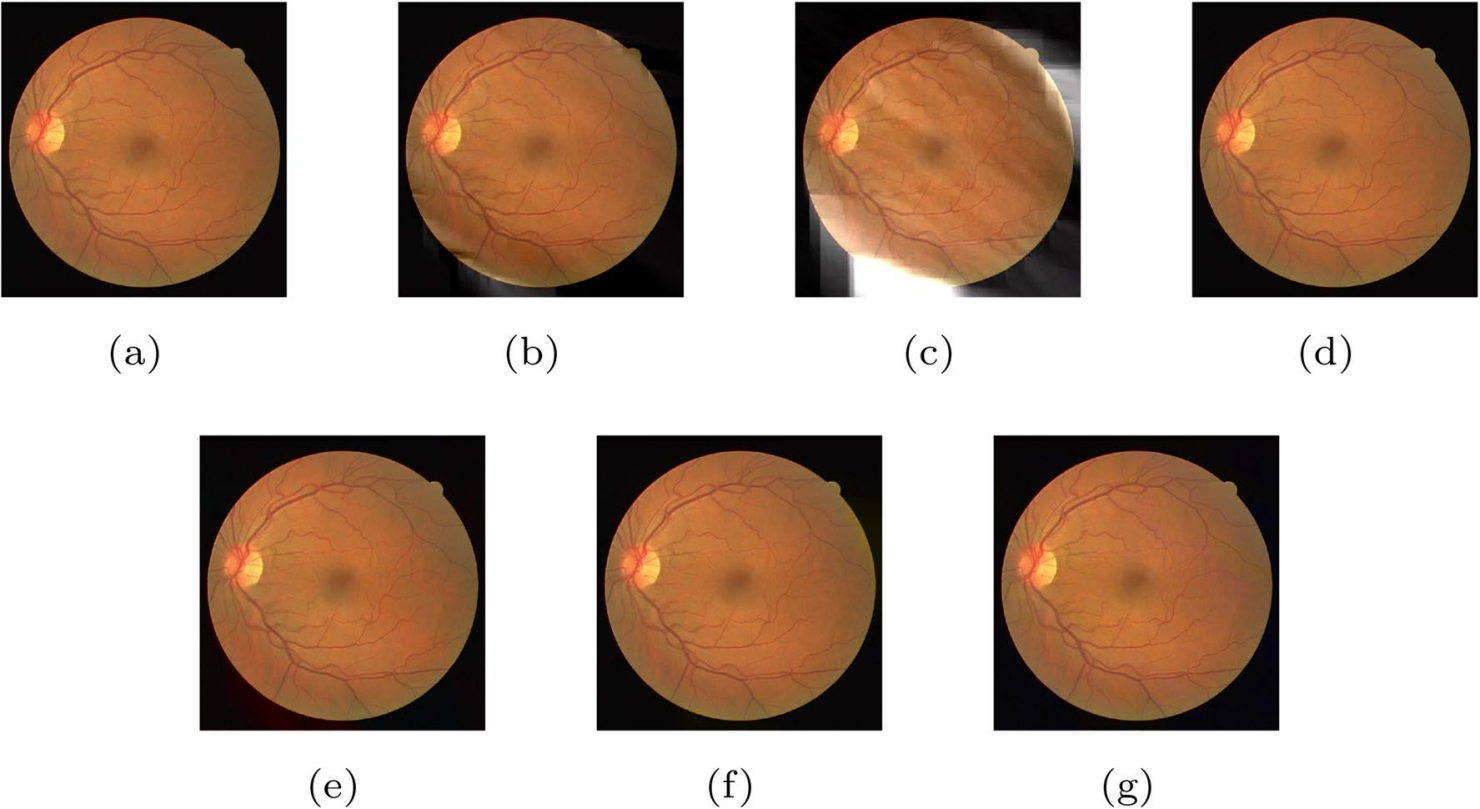
(**a**) The original image. (**b**) The reconstructed image with salt and pepper noise added to the component $Y_S$. (**c**) The reconstructed image with salt and pepper noise added to the component $Y_D$. (**d**) The reconstructed image with salt and pepper noise added to the components $U_S$. (**e**) The reconstructed image with salt and pepper noise added to the component $U_D$. (**f**) The reconstructed image with salt and pepper noise added to the component $V_S$. (**g**) The reconstructed image with salt and pepper noise added to the component $V_D$.

For the sake of clarity in the visualization, we show the reconstructed image with noise in Fig. 4. Each component is decoded after adding salt and pepper noise (mode I). Figure 4b and c have some distortion in the upper right and lower left regions compared with the original image. However, Fig. 3d–g are almost indistinguishable to the naked eye. This reveals the difference in the robustness of each component to such a kind of noise.

**Table 7:** The quality of the reconstructed image when salt and pepper noise is added to a component.

Noise	Metric $^1$	Component						
		$\varnothing$ $^2$	$Y_S$	$Y_D$	$U_S$	$U_D$	$V_S$	$V_D$
Mode I $^3$	PSNR	$\infty$	27.68	14.61	38.12	23.63	37.59	28.43
	SSIM	1	0.92	0.81	0.98	0.89	0.97	0.93
Mode II $^4$	PSNR	$\infty$	2.70	8.68	7.87	9.56	3.97	14.05
	SSIM	1	0.38	0.11	0.20	0.56	0.11	0.51

The experimental results are obtained on the DRIVE dataset.

$^1$The difference evaluation between the reconstructed image and the original image.

$^2$It means no loss of components.

$$^3$$Parameters: *a*=0, *b*=15, $$P_a$$=0.05, $$P_b$$=0.

$$^4$$Parameters: *a*=0, *b*=15, $$P_a$$=0, $$P_b$$=0.05.

### Gaussian noise

**Figure 5:**
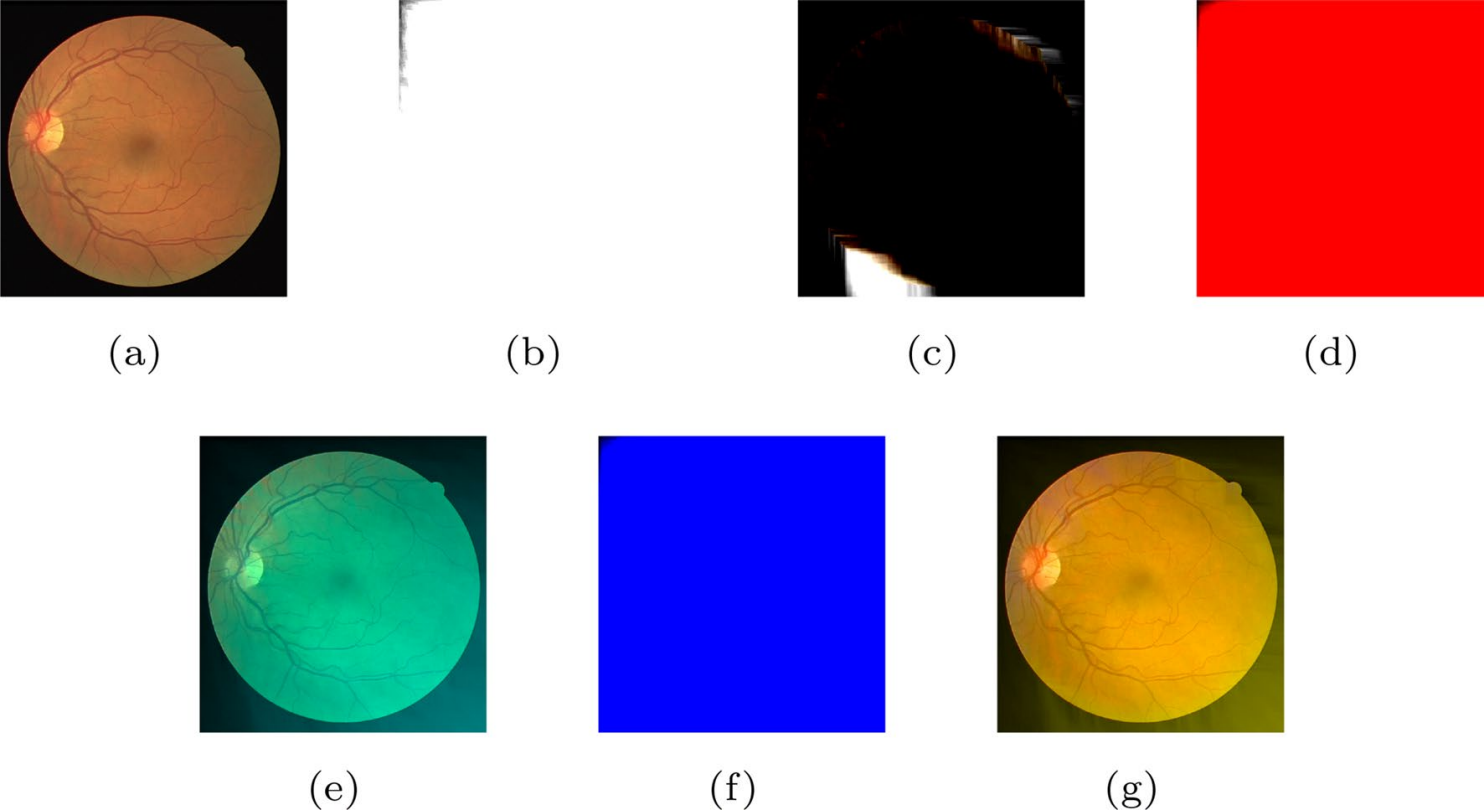
(**a**) The original image without any noise. (**b**) The reconstructed image with noise added to the component $Y_S$. (**c**) The reconstructed image with noise added to the component $Y_D$. (**d**) The reconstructed image with noise added to the components $U_S$. (**e**) The reconstructed image with noise added to the component $U_D$. (**f**) The reconstructed image with noise added to the component $V_S$. (**g**) The reconstructed image with noise added to the component $V_D$.

Gaussian noise models are used frequently in practice because of their mathematical tractability in both the spatial and frequency domains^36^. In fact, it is often used to model additive noise. The probability density function of Gaussian noise is given by

23 $$\begin{aligned} p(z) = {1 \over {\sqrt{2 \pi \sigma ^2}}} e^{ {-(z-\mu )^2} \over {2 \sigma ^2} } , \end{aligned}$$

where *u* is the mean value of *z*, and $\sigma$ is its standard deviation. On the one hand, we evaluate the impact of Gaussian noise from the image quality assessment metric and visual perception. Table 8 and Fig. 5 show the results. The shape layer is more sensitive to Gaussian noise than the detail layer, and the corresponding PSNR and SSIM values of the shape layer are lower. On the other hand, the visual difference is also more distinct.

**Table 8:** The quality of the reconstructed image when Gaussian noise is added to a component.

Image quality assessment metric $^1$	Component						
	$\varnothing$ $^2$	$Y_S$	$Y_D$	$U_S$	$U_D$	$V_S$	$V_D$
PSNR	$\infty$	2.67	7.37	7.85	7.82	3.96	14.37
SSIM	1	0.38	0.04	0.20	0.46	0.11	0.54

The experimental results are obtained on the DRIVE dataset. $u=0$, $\sigma =0.01$.

$^1$The difference evaluation between the reconstructed image and the original image.

$^2$It means no loss of components.

### Exponential noise

The PDF of exponential noise is given by

24 $$\begin{aligned} p(z)=\left\{ \begin{aligned}&c e^{-cz},~~{} & {} z\ge 0 \\&0,~~{} & {} z < 0 \\ \end{aligned} \right. \end{aligned}$$

where $c > 0$. The mean and variance of *z* are

25 $$\begin{aligned} \bar{z} = {1 \over c} \end{aligned}$$

and

26 $$\begin{aligned} \sigma ^2 = {1 \over c^2}. \end{aligned}$$

Table 9 shows the results when exponential noise is added to a component. It reaches the same conclusion that the detail layer is more robust to noise than the shape layer.

**Table 9:** The quality of the reconstructed image when exponential noise is added to a component.

Image quality assessment metric $^1$	Lost components						
	$\varnothing$ $^2$	$Y_S$	$Y_D$	$U_S$	$U_D$	$V_S$	$V_D$
PSNR	$\infty$	27.60	32.62	26.46	40.63	27.00	41.85
SSIM	1	0.88	0.93	0.86	0.98	0.86	0.98

The experimental results are obtained on the DRIVE dataset.

$^1$The difference evaluation between the reconstructed image and the original image.

$^2$It means no loss of components.

## Data transmission

In this section, we discuss communication issues when transmitting images with noisy channels. Specifically, the experiments focus on the distortion of the received data where the error-correcting code mechanism is not applied. It can more clearly reveal the robustness of each component in transmission. We consider two cases: binary symmetric channel and binary erasure channel.

### Binary symmetric channel (BSC)

**Figure 6:**
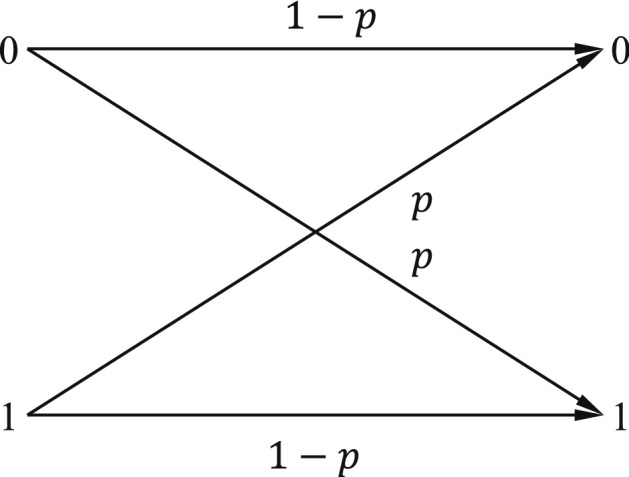
Binary symmetric channel.

In binary symmetric channel (BSC), the input symbols are completed with probability *p*, which is shown in Fig. 6. When an error occurs, a 0 is received as 1, and vice versa. The bits received do not reveal where the errors have occurred. In a sense, all the bits received are unreliable^2^. The information capacity of a binary symmetric channel with parameter *p* is

27 $$\begin{aligned} C = 1 -H(p)~~ \text {bits}. \end{aligned}$$

**Figure 7:**
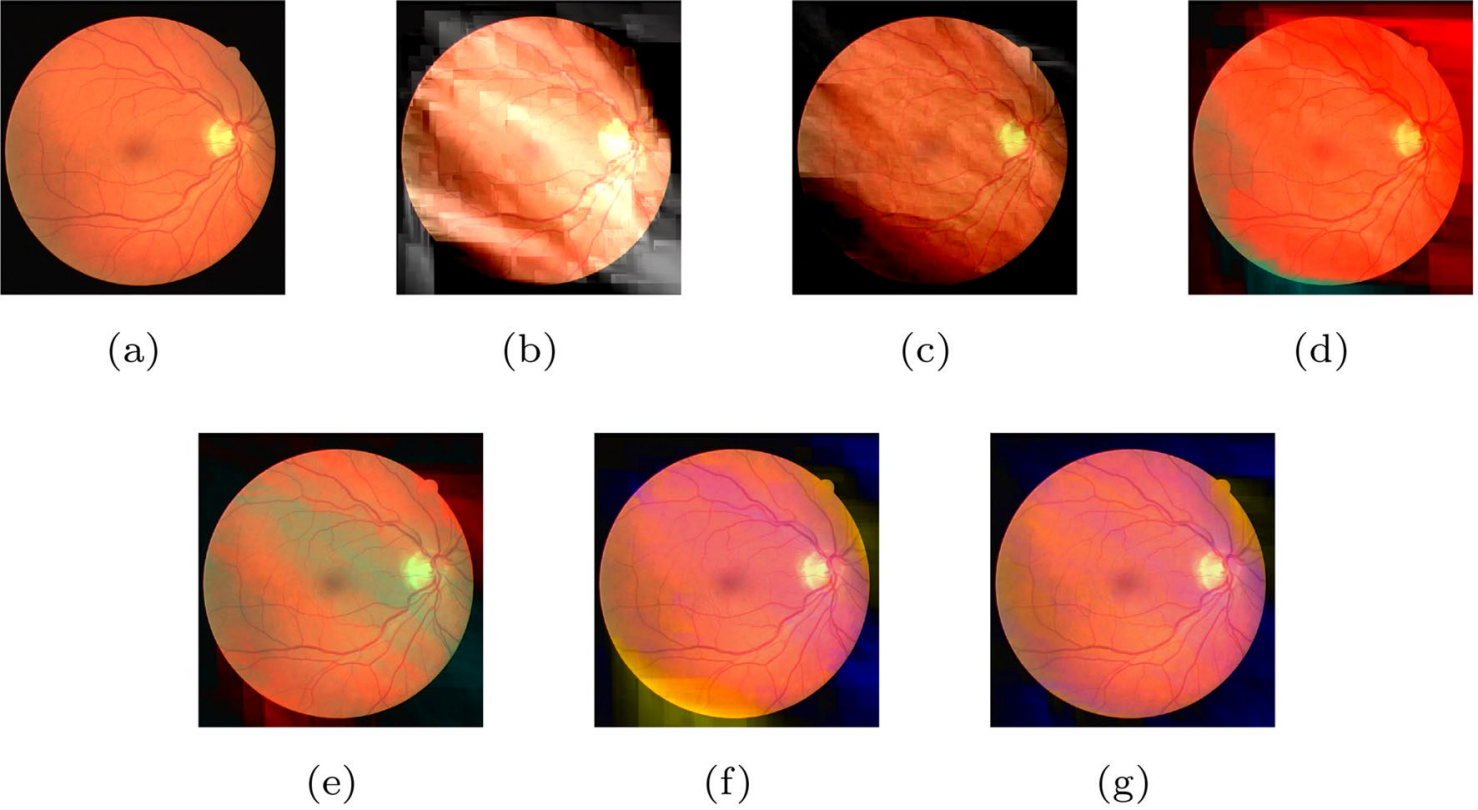
(**a**) The original image. (**b**) The reconstructed image when the component $Y_S$ is transmitted in the BSC. (**c**) The reconstructed image when the component $Y_D$ is transmitted in the BSC. (**d**) The reconstructed image when the component $U_S$ is transmitted in the BSC. (**e**) The reconstructed image when the component $U_D$ is transmitted in the BSC. (**f**) The reconstructed image when the component $V_S$ is transmitted in the BSC. (**g**) The reconstructed image when the component $V_D$ is transmitted in the BSC.

Table 10 reflects the quality of the reconstructed image when one component is transmitted through the binary symmetric channel and other components are transmitted through the noiseless channel. When $Y_S$ or $Y_D$ is transmitted in a noisy channel, the reconstructed image quality is lower than in the other four cases. This can also be seen from the visual point of view, Fig. 7b and c have serious distortion compared with the original image Fig. 7a. On the other hand, Fig. 7d–g have less distortion.

**Table 10:** The quality of the reconstructed image when one component is transmitted in the binary symmetric channel.

Image quality assessment metric	Components with noisy channel $^1$					
	$Y_S$	$Y_D$	$U_S$	$U_D$	$V_S$	$V_D$
PSNR	12.26	16.60	16.32	25.16	20.69	18.28
SSIM	0.59	0.57	0.67	0.87	0.80	0.73

The experimental results are obtained on the DRIVE dataset.

$$^1$$Parameters: *p*=0.01.

### Binary erasure channel (BEC)

**Figure 8:**
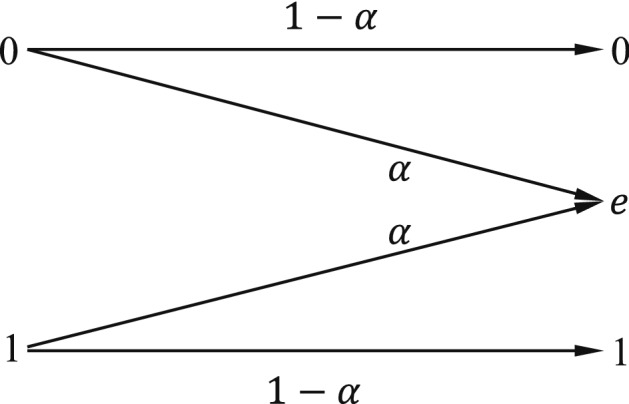
Binary erasure channel.

The analog of the binary symmetric channel in which some bits are lost (rather than corrupted) is referred to as the binary erasure channel. In this kind of channel, a fraction $\beta$ ($\beta$ > 0) of the bits is erased. As shown in Fig. 8, the receiver knows which bits have been erased. The binary erasure channel has two inputs and three outputs. Similarly, the information capacity of a binary erasure channel with parameter $\beta$ ($\beta$ > 0) is

28 $$\begin{aligned} C = 1 -\beta ~~ \text {bits}. \end{aligned}$$

Table 11 reflects the quality of the reconstructed image when a component is transmitted through the binary erasure channel and other components are transmitted through the noiseless channel. The results are similar to the BSC channel. When $Y_S$ or $Y_D$ is transmitted in a noisy channel, the reconstructed image quality is lower than in the other four cases. It reveals that $Y_S$ and $Y_D$ are more susceptible to noise than $U_S$, $U_D$, $V_S$ and $V_D$ during transmission.

**Table 11:** The quality of the reconstructed image when one component is transmitted in the binary erasure channel.

Image quality assessment metric $^1$	Components with noisy channel					
	$Y_S$	$Y_D$	$U_S$	$U_D$	$V_S$	$V_D$
PSNR	16.14	19.09	21.08	26.87	24.74	22.33
SSIM	0.71	0.67	0.80	0.89	0.86	0.80

The experimental results are obtained on the DRIVE dataset.

$$^1$$Parameters: $$\alpha$$=0.01.

**Figure 9:**
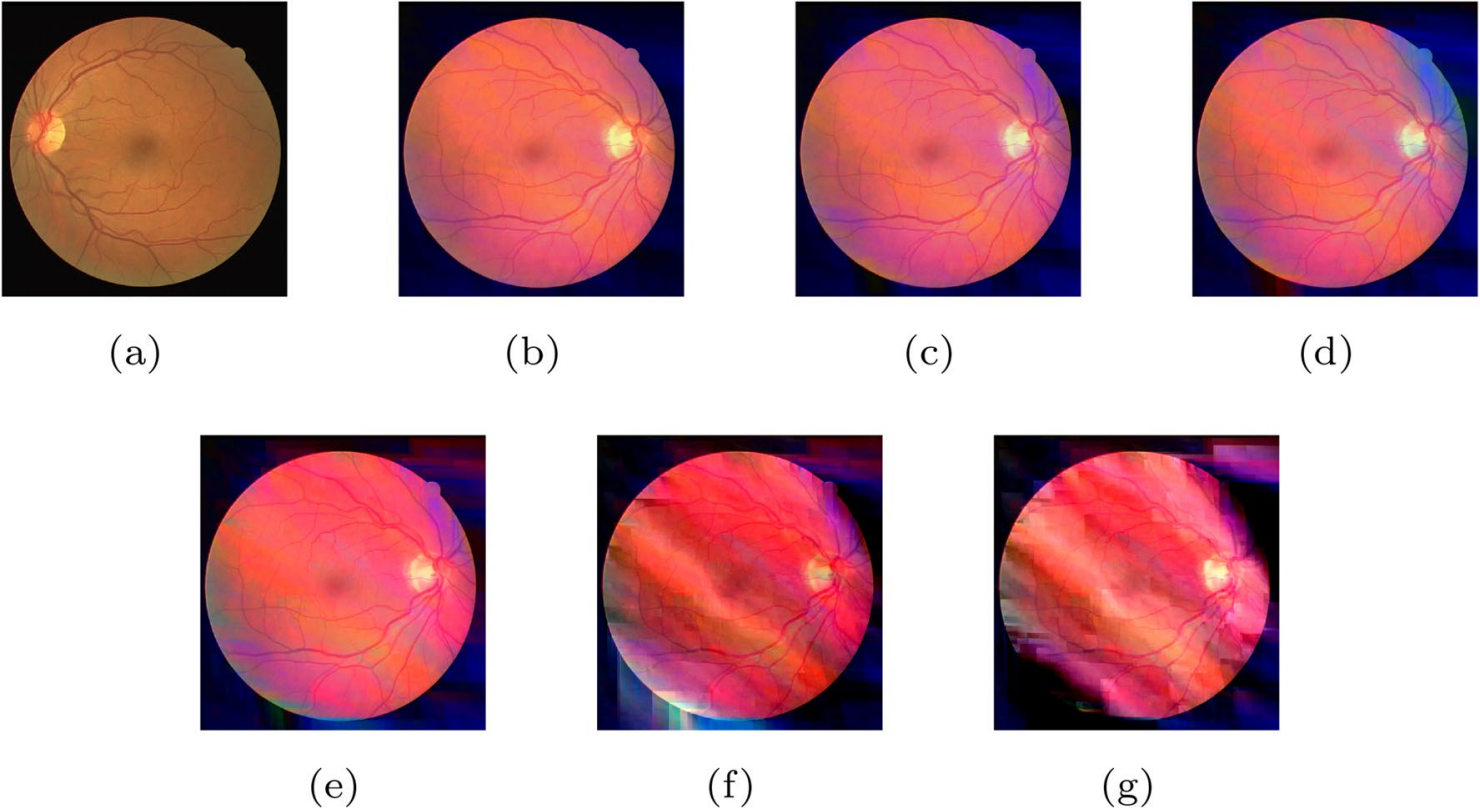
(**a**) The original image. (**b**) The reconstructed image when the components ($V_D$) are transmitted in the BEC. (**c**) The reconstructed image when the components ($V_D$, $V_S$) are transmitted in the BEC. (**d**) The reconstructed image when the components ($V_D$, $V_S$, $U_D$) are transmitted in the BEC. (**e**) The reconstructed image when the components ($V_D$, $V_S$, $U_D$, and $U_S$) are transmitted in the BEC. (**f**) The reconstructed image when the components ($V_D$, $V_S$, $U_D$ , $U_S$ and $Y_D$) are transmitted in the BEC. (**g**) The reconstructed image when the components ($V_D$, $V_S$, $U_D$, $U_S$, $Y_D$ and $Y_S$) are transmitted in the BEC.

In actual deployment, the condition of the channel changes at any time. Consequently, we need to explore the case where more components are transmitted with noisy channels. Figure 9 illustrates the visual effect of the reconstructed image as more components are transmitted through BEC. It can be clearly seen that the image quality is decreased as more components are disturbed by noise. Among them, Fig. 9d and e have the color changes, and Fig. 9f and g have the visible distortions. The data reported here appear to support the assumption that $Y_S$ and $Y_D$ represent the skeleton and structural information features, which are more susceptible to noise.

## Conclusion

This study sets out to do a comprehensive and practical analysis of image coding algorithms with soft compression. Compared with other diagrams, the novel algorithm has several distinctive characteristics: from hard to soft, from pixels to shapes, from fixed to random, and from coarse to fine. These changes bring the efficiency and effectiveness of soft compression, making it suitable for image compression tasks in the era of big data. In this paper, the system was deployed in several scenarios, consisting of mutual information measure analysis, function evaluation of each component, noise robustness, and data transmission.

The second aim of this study was to investigate the effects of each component on the complete image. The shape and detail layer jointly form an image from coarse to fine. Specifically, $Y_S$ and $Y_D$ represent the skeleton and structural features of an image, respectively. They contribute much more to visual perception than other components. Moreover, $U_S$ and $V_S$ reflect the spatial hierarchy feature, and $U_D$ and $Y_D$ reveal the chroma feature. The insights gained from this work may be of assistance to joint source-channel coding.

The present study contributes to our understanding of soft compression. It provides the first comprehensive investigation of the algorithm and establishes a quantitative framework for evaluating each component on images. Although it focuses on several scenarios, the findings may well have a bearing on general image coding methods. Moreover, the study provides experimental data and side information for the actual deployment of the system. Continued efforts are needed to make soft compression more prone to rapid and drastic changes. All these observations will be expected to provide more insights into the future multi-media semantic communications and find promising applications in the metaverse and digital twins, etc.

## Data Availability

The code of the soft compression algorithm is available from the corresponding author on reasonable request and can also be found at https://github.com/ten22one/Soft-compression-algorithm-for-multi-component-image.
